# Developing Age-Friendly Communities: Comparative Research on the Role of Social Infrastructure and Inclusion

**DOI:** 10.1093/geroni/igaa057.2387

**Published:** 2020-12-16

**Authors:** Chris Phillipson, Jarmin Yeh, Chris Phillipson

## Abstract

Aging experiences are often framed by the forms in which communities take shape. In turn, the material forms of communities provide the conditions in which aging experiences and processes are produced. These multidirectional pathways of influence between micro-macro contexts comprise an assemblage of networks that usually go unnoticed, except on breakdown. Applying Leigh-Star’s (1999) charge to study these networks as infrastructure, we examine their “invisible work” in the ecological analysis of aging in community. This symposium presents research using community-embedded methods to illuminate how paradoxical forms of transparent and opaque infrastructure facilitate and mediate interactions between people, things, and the environmental shapes that hold these interactions. Yarker’s comparative case analysis of two Manchester neighborhoods highlight the critical importance of social infrastructure in supporting the diversity of relations needed to develop age-friendly communities and new forms of community action amongst older people. Grove’s qualitative and geo-spatial approaches demonstrate how planned and unplanned social interactions, and variety of social infrastructural forms, contribute positively and negatively to ageing well for older people in Dublin. Simon-Rusinowitz’s three-part intervention addresses unmet needs of older Baltimore residents in a low-income community; finding, in part-one, that housing is a platform for providing health and social services needed to age successfully. Yeh compares the conceptual frames undergirding age-friendly community initiatives with expert knowledges and lay perspectives of older San Franciscans, revealing tension between the rational dreams and material realities of aging in the community. These presentations contribute to gerontological discourse, providing new insights for policy and practice considerations.

